# ‘Dunbar's number’ deconstructed

**DOI:** 10.1098/rsbl.2021.0158

**Published:** 2021-05-05

**Authors:** Patrik Lindenfors, Andreas Wartel, Johan Lind

**Affiliations:** ^1^ Institute for Futures Studies, Box 591, 101 31 Stockholm, Sweden; ^2^ Centre for Cultural Evolution, Stockholm University, Sweden

**Keywords:** phylogenetic comparative studies, social evolution, brain evolution, primates, mammals

## Abstract

A widespread and popular belief posits that humans possess a cognitive capacity that is limited to keeping track of and maintaining stable relationships with approximately 150 people. This influential number, ‘Dunbar's number’, originates from an extrapolation of a regression line describing the relationship between relative neocortex size and group size in primates. Here, we test if there is statistical support for this idea. Our analyses on complementary datasets using different methods yield wildly different numbers. Bayesian and generalized least-squares phylogenetic methods generate approximations of average group sizes between 69–109 and 16–42, respectively. However, enormous 95% confidence intervals (4–520 and 2–336, respectively) imply that specifying any one number is futile. A cognitive limit on human group size cannot be derived in this manner.

## 1. Introduction

‘Dunbar's number’ is the notion that there exists a cognitive limit on human groups of about 150 individuals. [1,2] This because ‘[t]o maintain group cohesion, individuals must be able to meet their own requirements, as well as coordinate their behaviour with other individuals in the group. They must also be able to defuse the direct and indirect conflicts that are generated by foraging in the same space’. [3] According to the hypothesis, since the neocortex is commonly believed to play a crucial role in handling social relationships [4], its size should set an upper limit on the number of stable social relationships that primate brains can keep track of and maintain.

The number 150 was established by extrapolating a regression line describing the relationship between group size and relative neocortex size in primates, to humans. [1,2,5,6] That there exists a correlation between group size and relative neocortex size has been replicated in several studies (e. g. [7–14]), though in some cases only for female primates [15,16], but often not finding a significant relationship (making an estimate of Dunbar's number unachievable) [11,14,17,18]. However, the replication studies are of somewhat limited value as most studies have used the same brain data. [14] Additional studies have been made using similar reasoning, but analysing relative brain size instead of relative neocortex size (e. g. [9,19,20]).

The expected human group size of 150 has been substantiated by observations of human communities with group sizes ranging between 100 and 200, including hunter–gatherer communities, military units, businesses, 18th-century and Neolithic villages, information from the Domesday Book [2] and Christmas card networks [6].

‘Dunbar's number’ is often cited^1^, has had great impact in popular culture (e.g. it featured prominently in Malcolm Gladwell's book *Tipping point* [21]) and has had consequences such as the Swedish Tax Authority restructuring their offices to stay within the 150-person limit [22], with the implicit but hopefully unintended assumption that their employees have neither family nor friends outside work.

Attempting to make a decisive deconstruction of the empirical basis of Dunbar's number, we here perform Bayesian and generalized least-squares (GLS) phylogenetic comparative analyses on larger datasets of the relationship between group size and both relative brain and relative neocortex sizes, and then extrapolate from these relationships to arrive at an updated estimate of the cognitive limit on human group size, including confidence intervals.

## 2. Methods

Volumes on brain components were averaged from three sources [18,23,24]. Only measurements of individuals where both brain volume and neocortex volume were available were included in analyses of neocortex size. However, one of the datasets contains brain region measurements that differ significantly from the others [25]. For this reason, we replicated our analyses using data from only the most carefully collected dataset [18]. These results are presented in the electronic supplementary material, appendix S1 (electronic supplementary material, tables S2 and S3). The results from these analyses do not differ in any meaningful way from those provided in the main paper, however. We also present results from measurements of endocranial volumes with corresponding estimates of body mass [26] and replicate all analyses also on anthropoid primates only (electronic supplementary material, tables S1 and S3). Average brain weights, body weights and group size data were taken from one recent study [20], complemented with additional group size data where available [26]. Measures on the human brain [27] and body mass [28] were collected separately. Consensus phylogenies for each dataset were obtained from the 10kTrees website [29]. All brain measures were log-transformed prior to analysis, except when using ratios, that instead were arcsine-square root transformed.

All analyses were executed in R using the packages NLME [30], APE [31], MASS [32] and BRMS [33]. We used Bayesian multilevel models with varying intercepts over species specified by a covariance matrix and phylogenetic generalized least-squares (PGLS) regressions throughout. In all Bayesian analyses, group size was modelled as a continuous gamma distribution and not discrete Poisson because available data are averages calculated from different sources. Results for the Bayesian analyses will differ some for each time running the analyses and are only fully reproducible using the same machine. For the Bayesian analyses, uncertainty associated with phylogeny was added by taking random samples from the posterior distribution of the existing random effects, centred around zero and used for calculating confidence intervals. Ninety-five per cent of confidence intervals for GLS were calculated without phylogenetic effects. As electronic supplementary material, appendix S2, we include the R-code and resulting output.

## 3. Results

Our results (table 1) reveal that estimates of expected human group sizes vary depending on method and variable choice (Bayesian approximations between 69.2 and 108.6 and GLE approximations between 16.4 and 42.0). Note that these estimates (as was true for Dunbar's original estimated group size) are averages, not estimates of upper bounds. If an upper constraint from this type of statistical reasoning was to be determined, a better approach would be to specify the upper boundary of the 95% confidence interval. As is shown in table 1, however, 95% confidence intervals yield enormous variation in their estimates, 3.8–520.0 and 2.1–336.3, respectively, and thus indicate upper limits far exceeding 150 in almost all cases.

**Table 1.** Estimates of human group sizes from phylogenetic comparative analyses of relative primate brain and neocortex sizes. Due to the probabilistic nature of Bayesian statistical tests, replications of these analyses will yield similar but not necessarily identical results.

model	estimated human group size	lower 95% bound	upper 95% bound
*Bayesian analyses*			
group size^a^ ∼ neocortex^b^ + rest of brain^b^ (*n* = 71)	69.2	3.8	292.0
group size^a^ ∼ neocortex^b^/rest of brain^b^ (*n* = 71)	108.6	4.6	520.0
group size^a^ ∼ brain weight^c^ + body weight^c^ (*n* = 142)	79.8	4.1	329.9
*generalized least-squares*			
group size^a^ ∼ neocortex^b^ + rest of brain^b^ (*n* = 71)	16.4	2.1	127.7
group size^a^ ∼ neocortex^b^/rest of brain^b^ (*n* = 71)	42.0	5.2	336.3
group size^a^ ∼ brain weight^c^ + body weight^c^ (*n* = 142)	23.6	3.2	175.6
*Bayesian analyses*			
group size^a^ ∼ brain volume^d^ + body weight^d^ (*n* = 126)	79.1	4.0	335.4
*generalized least-squares*			
group size^a^ ∼ brain volume^d^ + body weight^d^ (*n* = 126)	23.8	3.2	177.8

^a^DeCasien *et al*. [18] & Kappeler & Heymann [34].

^b^Navarrete *et al*. [24] & DeCasien *et al*. [18].

^c^DeCasien *et al*. [20].

^d^Isler *et al*. [26].

The best model to test the hypothesis, given Dunbar's original formulation (a cognitive limit on group size deduced from relative neocortex size) and the current state-of-the-art of comparative phylogenetic studies, is the Bayesian analyses of group size using the volume of the neocortex, with the volume of the rest of the brain included as a covariate. The estimate from this analysis indicates a human group size average of 69.2 individuals, with a 95% confidence interval ranging from 3.8 to 292.0 individuals. This is not very informative. We also provide results from other methods and variable choices, for comparison, both in table 1 and in electronic supplementary material, appendix S1. These do not provide estimates with more enlightening estimated confidence intervals, though one of our estimates actually came close to 150 (electronic supplementary material, appendix S1; table 1, top row: 134.0), but again, the confidence intervals were enormous (7.0 to 583.2).

## 4. Discussion

Most research on primate social evolution has not concerned cognitive limitations but instead generally focused on the so-called ‘socio-ecological model of primate social evolution’ where primate group size mainly is determined by socio-ecological factors having to do with foraging and predation, infanticide and sexual selection—not on brain or neocortex size. According to this model of social evolution, females go where it is safe and where there are resources, while males go where the females are [35–38].

Further, it is easily observed that human brains function differently from those of other primates [38–42], as is evidenced by the existence of cumulative cultural evolution resulting in marvels such as Stockholm, symphonies and science [43–45]. This was concisely summarized by de Ruiter *et al*. [22] in their examination of Dunbar's number: ‘Dunbar's assumption that the evolution of human brain physiology corresponds with a limit in our capacity to maintain relationships ignores the cultural mechanisms, practices, and social structures that humans develop to counter potential deficiencies’.

Also, researchers have disputed the empirical observation of mean human group sizes approximately averaging around 150 persons, presenting empirical observations of group sizes indicating a wide variety of other numbers [46–53]. Thus, ecological research on primate sociality, the uniqueness of human thinking and empirical observations all indicate that there is no hard cognitive limit on human sociality. Our reanalysis provides the last piece of evidence needed to disregard Dunbar's number.

In summary, extrapolating human cognitive limits from regressions on non-human primate data is of limited value for both theoretical and empirical reasons. It is our hope, though perhaps futile, that this study will put an end to the use of ‘Dunbar's number’ within science and in popular media. ‘Dunbar's number’ is a concept with limited theoretical foundation lacking empirical support.

## Endnotes

^1^ Dunbar's original paper has over 2500 citations on Google Scholar.

## Data accessibility

Data used in this study have been deposited in the Dryad Digital Repository: https://doi.org/10.5061/dryad.ghx3ffbnn [54].
